# The EIIIA domain from astrocyte‐derived fibronectin mediates proliferation of oligodendrocyte progenitor cells following CNS demyelination

**DOI:** 10.1002/glia.22748

**Published:** 2014-08-25

**Authors:** Josephine M. J. Stoffels, Dick Hoekstra, Robin J. M. Franklin, Wia Baron, Chao Zhao

**Affiliations:** ^1^ Department of Cell BiologyUniversity of Groningen, University Medical Center GroningenThe Netherlands; ^2^ Wellcome Trust‐Medical Research Council Cambridge Stem Cell Institute and Department of Clinical NeurosciencesUniversity of CambridgeUnited Kingdom

**Keywords:** fibronectin, astrocyte, oligodendrocyte, remyelination, multiple sclerosis

## Abstract

Central nervous system remyelination by oligodendrocyte progenitor cells (OPCs) ultimately fails in the majority of multiple sclerosis (MS) lesions. Remyelination benefits from transient expression of factors that promote migration and proliferation of OPCs, which may include fibronectin (Fn). Fn is present in demyelinated lesions in two major forms; plasma Fn (pFn), deposited following blood‐brain barrier disruption, and cellular Fn, synthesized by resident glial cells and containing alternatively spliced domains EIIIA and EIIIB. Here, we investigated the distinctive roles that astrocyte‐derived Fn (aFn) and pFn play in remyelination. We used an inducible Cre‐lox recombination strategy to selectively remove pFn, aFn or both from mice, and examined the impact on remyelination of toxin‐induced demyelinated lesions of spinal cord white matter. This approach revealed that astrocytes are a major source of Fn in demyelinated lesions. Furthermore, following aFn conditional knockout, the number of OPCs recruited to the demyelinated lesion decreased significantly, whereas OPC numbers were unaltered following pFn conditional knockout. However, remyelination completed normally following conditional knockout of aFn and pFn. Both the EIIIA and EIIIB domains of aFn were expressed following demyelination, and *in vitro* assays demonstrated that the EIIIA domain of aFn mediates proliferation of OPCs, but not migration. Therefore, although the EIIIA domain from aFn mediates OPC proliferation, aFn is not essential for successful remyelination. Since previous findings indicated that astrocyte‐derived Fn aggregates in chronic MS lesions inhibit remyelination, aFn removal may benefit therapeutic strategies to promote remyelination in MS. GLIA 2015;63:242–256

## Main Points

- We investigated the roles of cellular and plasma fibronectin in remyelination, and found that astrocyte‐derived fibronectin mediates oligodendrocyte progenitor cell proliferation in a toxin‐induced demyelination model, likely via the EIIIA domain.

## Introduction

Multiple sclerosis (MS) is a central nervous system (CNS) disease, of which inflammation, demyelination, and axonal loss are the main pathological features. Regeneration of myelin (remyelination) involves proliferation and migration of activated oligodendrocyte progenitor cells (OPCs) to demyelinated lesions, and their subsequent differentiation into myelinating oligodendrocytes (Franklin and ffrench‐Constant, 2008; Zawadzka et al., 2010). The extent of remyelination in MS is variable, but often is insufficient to prevent chronic axonal loss (Franklin et al., 2012; Patrikios et al., 2006). Promoting endogenous remyelination provides a means to reduce axonal degeneration and thereby slow progression of clinical disability (Duncan et al., 2009; Franklin et al., 2012).

Fibronectin (Fn) is an extracellular matrix (ECM) protein, that is essential for embryonic development (George et al., 1993). In healthy adults, Fn is continuously produced by hepatocytes and circulates in the plasma. Fn is absent from the healthy CNS, but expressed following injury, which includes demyelination. Plasma Fn (pFn) leaks across a disrupted blood‐brain barrier (Satoh et al., 2009; Sobel and Mitchell, 1989; van Horssen et al., 2005), and cellular Fn (aFn) is primarily synthesized by astrocytes (Hibbits et al., 2012; Stoffels et al., 2013a), but also by microglia/macrophages and endothelial cells (Stoffels et al., 2013a). In contrast to pFn, cellular Fn may contain alternatively spliced domains, named EIIIA, EIIIB, and the V‐region in rodents (EDA, EDB, and IIICS, respectively, in humans; Paul et al., 1986; Schwarzbauer et al., 1987). *In vitro*, pFn stimulates migration and proliferation of OPCs at low growth factor levels via αv integrin receptors, the only integrin receptors for Fn present on OPCs (Baron et al., 2002; Blaschuk et al., 2000; Milner and ffrench‐Constant, 1994; Milner et al., 1996). The expression of both Fn and αv integrins is transiently increased in demyelinated lesions (Stoffels et al., 2013a; Zhao et al., 2009). Therefore, Fn may contribute to remyelination by promoting OPC recruitment. However, at later stages of oligodendrocyte maturation, removal of Fn is required for remyelination to proceed to completion, since Fn inhibits myelin sheet formation (Buttery and ffrench‐Constant, 1999; Maier et al., 2005; Siskova et al., 2006, 2009). In addition, Fn aggregates impair OPC differentiation *in vivo*, and likely contribute to remyelination failure in MS (Stoffels et al., 2013a).

Here, we explored the role of Fn in more depth by asking what distinctive roles the two Fn variants, pFn and astrocyte‐derived Fn (aFn), play in remyelination. Using an inducible Cre‐lox recombination strategy to selectively remove pFn, aFn or both (Hirrlinger et al., 2006; Sakai et al., 2001) in combination with a well‐established model of CNS de‐ and remyelination (Blakemore and Franklin, 2008), we found that conditional knockout of aFn, but not pFn, reduced the density of OPCs. *In vitro* analysis revealed that this was likely mediated by the EIIIA domain, which mediates OPC proliferation on aFn at sufficient growth factor levels. However, although conditional knockout of aFn was associated with reduced OPC numbers following demyelination, it was not sufficient to affect the remyelination outcome. The translational implication of our data therefore is that elimination of aFn may be amenable in MS to prevent the formation of remyelination‐inhibiting Fn aggregates. This will likely be beneficial in promoting endogenous remyelination (Stoffels et al., 2013a).

## Materials and Methods

### Mice

Mice were housed under standard conditions. All experiments were performed in compliance with United Kingdom Home Office regulations. Plasma Fn (pFn) inducible, conditional knockout mice (hereafter referred to as pFn^cKO^) were a kind gift from Dr. R. Fässler, Max Planck Institute for Biochemistry, Martinsried, Germany. Inducible, conditional knockout (hereafter referred to as “conditional knockout”) of pFn was created as described (Sakai et al., 2001). Briefly, floxed Fn mice were crossed with mice expressing Cre recombinase under the control of the polyinosinic‐polycitidic acid (poly I:C) responsive Mx promoter (Mx‐Cre). On Cre‐mediated recombination at the *loxP* sites, the start codon, signal sequence and the exon/intron border of exon 1 are removed from the Fn gene to generate the null allele (Sakai et al., 2001). Cre‐mediated recombination was induced in hepatocytes from the 6‐week old mice carrying Mx‐Cre by two intraperitoneal injections of poly I:C (GE Healthcare, Amersham, UK) with a 48 h interval as previously described (Sakai et al., 2001). Wild type (WT) control mice received vehicle only (phosphate‐buffered saline; PBS). Mice were subjected to lysolecithin‐induced demyelination at 2–3 weeks following induction of the knockout.

Conditional knockout mice devoid of aFn (astrocyte Fn; aFn^cKO^) were created by crossing Fn floxed mice with mice expressing Cre recombinase driven by human glial fibrilary acid protein (GFAP), with its nucleus translocation controlled by a modified estrogen receptor (hGFAP‐CreERT2; Hirrlinger et al., 2006). The hGFAP‐CreERT2 mice were a kind gift from Dr. F. Kirchhoff, Max Planck Institute of Experimental Medicine, Goettingen, Germany. To induce conditional knockout of Fn from astrocytes, tamoxifen (100 mg/kg in corn oil, Sigma‐Aldrich, Gillingham, UK) was injected intraperitoneally daily for 5 consecutive days, starting from 10 days prior to demyelination (Hirrlinger et al., 2006; Leone et al., 2003). The littermate WT control group was injected with corn oil.

Compound astrocyte and pFn conditional knockout (a + pFn^cKO^) was achieved by breeding mice heterozygous for MxCre and hGFAP‐CreERT2, and homozygous for the floxed Fn gene. The induction protocol for these mice was the combination of that described for single conditional knockout strains above.

### Lysolecithin‐Induced Demyelination of the Spinal Cord and Tissue Processing

Surgery and tissue processing were performed as described (Zhao et al., 2006). Briefly, mice at about 9–10 weeks old were anaesthetized with isoflurane, and spinal cord lesions were created by direct injection of 1 µL 1% lysolecithin into the ventral funiculus through a gap between two thoraco‐lumbar vertebrae. In the conditional knockout mice, lesions were induced 1–2 weeks after completing the induction protocol. Blood samples were obtained for isolating plasma from the tail at the time of lesion, and collected in citrate‐dextrose solution (Sigma‐Aldrich, Dorset, UK), then stored at −80°C until use.

At the designated time post lesion, mice were euthanized by intraperitoneal injection of pentobarbital followed by appropriate protocols of tissue processing. For immunohistochemistry and *in situ* hybridization, mice were perfusion fixed with 4% phosphate‐buffered paraformaldehyde (PFA) solution via the left ventricle, after which the dissected spinal cord containing the lesions was either directly frozen at −80°C for later RNA extractions, or treated with 20% sucrose in PBS overnight. Cords were embedded in OCT (Thermo Fisher Raymond Lamb, Loughborough, UK), and cut in coronal sections at 12 µm thickness. These sections were mounted on poly‐l‐lysine (PLL)‐coated slides (Polysciences Europe GmbH Eppelheim, Germany) and stored at −80°C until further use. For resin embedding and semi‐thin sectioning, mice were perfused with 4% phosphate‐buffered gluteraldehyde and subjected to a standard resin embedding process (Zhao et al., 2006). Semi‐thin sections of 1 µm were cut and stained with alkaline toluidine blue. Ranking analysis on semi‐thin sections of remyelinated lesions was performed by two independent, blinded researchers as described (Ibanez et al., 2004).

### Glial Cell Cultures

Primary glial cell cultures were generated from 1 to 2 day old Wistar rats (Harlan, the Netherlands) as previously described (Baron et al., 2002; Bsibsi et al., 2012). Briefly, after 10–12 days in culture on PLL (5 µg/mL, Sigma‐Aldrich, St. Louis, MO) coated flasks, OPCs and astrocytes were isolated via a shake‐off procedure (McCarthy and de Vellis, 1980). Contaminating microglia were removed by shaking the flasks at 150 rpm for 1 h at 37°C on an orbital shaker. Subsequently, flasks were shaken at 240 rpm overnight at 37°C. Floating OPCs were further purified by differential adhesion. Then, OPCs were cultured in defined Sato medium (Maier et al., 2005) on appropriate cell culture plastics for the different assays (see below). Purity of the OPC cultures was routinely assessed by immunocytochemistry for Olig2 (1:1000; Millipore, Billerica, MA) and cultures used were >97% pure.

To obtain astrocytes, a subsequent overnight shake off was performed at 240 rpm and the remaining astrocyte monolayer was removed by trypsin. Astrocytes were cultured in T162 flasks (Corning Costar, Lowell, MA) in DMEM (Life Technologies, Paisley, UK) containing 10% fetal calf serum (FCS; Bodinco, Alkmaar, The Netherlands) and antibiotic supplements (Life Technologies), and passaged once before experimental use. Regular immunocytochemistry for glial fibrillary acidic protein (GFAP; Millipore; 1:500) was performed to assure sufficient purity of the astrocyte cultures (>97%).

### Plasma and Astrocyte‐Derived Fibronectin Coatings

To obtain aFn, astrocytes were cultured on 10 cm dishes (Corning) for 48 h, followed by water‐lysis at 37°C. The remaining astroglial matrix was scraped in sterile PBS containing Complete Mini protease inhibitor cocktail (Roche Diagnostics, Mannheim, Germany), followed by protein concentration determination with Bio‐Rad DC protein assay (Bio‐Rad Laboratories, Hercules, CA) using bovine serum albumin (BSA) as standard. Then, 8‐well chamber‐slides were incubated with 8 µg pFn from serum (Sigma‐Aldrich) or 8 µg astroglial matrix protein (“aFn”) per well in PBS for 3 h at 37°C. Similarly, both sides of membranes on transwell microchambers (Becton‐Dickinson Labware, Bedford, MA) were coated with 10 µg of pFn or aFn per well, and 3.5 µg pFn or aFn was used per well of a 96‐wells plate. When indicated, Fn coatings were incubated with functional blocking antibodies (2 µg/mL) for 1 h at 37°C, followed by a gentle wash with PBS and seeding of OPCs. Blocking antibodies used were: mouse anti‐Fn IST9 (directed against the EIIIA domain, Abcam, Cambridge, UK) and mouse anti‐Fn C6 (directed against the EIIIB domain, Abcam). Notably, the aFn preparation also contains other astroglial matrix proteins, such as laminin (Liesi et al., 1983, 1984) and chondroitin sulphate proteoglycans (Lau et al., 2012), but although these proteins may affect OPC adhesion, proliferation and migration also (Hu et al., 2009; Lau et al., 2012; Milner et al., 1996), EIIIA and EIIIB are exclusively present in aFn.

### SDS‐PAGE and Western Blotting

Equal amounts of plasma from pFn^cKO^ and a + pFn^cKO^ mice and their controls were mixed with standard SDS sample buffer, heated for 10 min at 95°C, and separated by electrophoresis on SDS‐PAGE gels (8%, Expedeon, Cambridge, UK) for 1 h at 150 V. Protein was transferred to a PVDF membrane (Invitrogen, Paisly, UK) using a wet blotting system (Expedeon, Cambridge, UK) and according glycine‐Tris‐methanol buffer. After three washes with 0.1% Tween‐20 in PBS, membranes were blocked with 5% non‐fat milk solution and incubated with a rabbit anti‐Fn antibody (Millipore, Watford, UK; 1:10,000) in blocking buffer, overnight at 4°C. Membranes were washed, and incubated with HRP‐conjugated anti‐rabbit antibody (Roche, Lewes, UK; 1:1000) in washing buffer. Signals were detected using enhanced chemiluminescence plus (ECL plus; GE Healthcare, Amersham, UK) followed by exposure on suitable X‐ray film (Thermo Scientific, Rockford, IL). For detection of mouse IgG as a control, membranes were washed and incubated with biotinylated donkey antimouse IgG antibody (Jackson ImmunoResearch Laboratories, Newmarket, UK; 1:1000) for 1 h, after which the Vectastain ABC Elite kit (Vector Laboraties, Peterborough, UK) was used according to the manufacturer's instructions, and signals were developed as described above.

### Immunochemistry

Frozen spinal cord sections were air dried for ∼1 h and immunohistochemistry was performed as described (Stoffels et al., 2013a) using antibodies against Fn (Millipore; 1:1000), Olig2 (Millipore, 1:1000; R&D Systems, Abingdon, UK; 1:200), Sox2 (Santa Cruz Biotechnology Inc, Dallas, TX; 1:500), KI67 (Abcam; 1:1000), and Iba1 (Abcam; 1:500). Antigen retrieval was performed before immunostaining with Olig2, using target retrieval solution (DAKO, Ely, UK) at 95°C for 20 min. Immunochemistry on coated aFn was performed as described (Stoffels et al., 2013a), using antibodies against Fn (1:50), IST9 (1:50), and C6 (1:50). Secondary antibodies used were appropriate Alexa Fluor© (488 or 594)‐conjugated secondary antibodies (Invitrogen; 1:500).

For immunohistochemistry using primary antibodies generated in mice on mouse tissue, a modified protocol was used. This protocol was applied for primary antibodies against Nkx2.2 (Developmental Studies Hybridoma Bank, Iowa, IA; 1:100), CC1 (Millipore; 1:100) and Aldh1L1 (NeuroMab, Davis, CA; 1:100) and secondary antibodies used were Alexa Fluor© 488‐conjugated‐anti IgG2b (for Nkx2.2 and CC1) or ‐anti IgG1 (for Aldh1L1; Invitrogen; 1:400). Sections were first permeabilized with 1% Triton‐X‐100 in 0.05 M Tris‐buffered saline (TBS) for 30 min. Then, sections were blocked for 30 min in TBS with 10% normal goat serum (NGS) and 0.25% Triton‐X‐100, followed by another blocking step for 1 h using mouse‐on‐mouse Ig solution (Vector Laboratories) according to the manufacturer's instructions. Primary antibodies were appropriately diluted in TBS containing 2% NGS and 0.3% Triton‐X‐100, and applied for 1 h. Secondary antibodies were appropriately diluted in TBS with 1% NGS, 0.1% Triton‐X‐100 and DAPI (1 µg/mL), and applied for 15 min, followed by a 10 min incubation in 0.1% Sudan Black in 70% ethanol. All incubations were at room temperature. After each step, sections were washed in 0.05 M TBS with 0.1% Tween‐20 (VWR, Lutterworth, UK) 3 times 2 min.

Images from immunohistochemistry with either protocol were acquired using a Zeiss Observer A1 fluorescent microscope, and images from immunochemistry on coatings were acquired using a Zeiss Axioskop 2 microscope with Leica Application Suite V3 software. For counting cell numbers, in each animal 3 demyelinated lesion levels were selected, spanning the centre of lesions, at ∼120 µm distance from each other. The outline of each lesion was defined based on the increase in DAPI cellularity inside the lesion using Zeiss Axovision 4.8 software, which corresponds to the demyelinated area as stained for with Sudan Black (Stoffels et al., 2013a). The numbers of marker‐positive cells inside the lesions were manually counted three times and averaged for each lesion. There were 4–5 mice per group. To allow for quantitative comparison, sections were stained in parallel and image acquisitions were performed on the same day using identical camera settings. Individual Fn‐ and Iba1‐immunereactive cells could not be discerned reliably, hence these were quantified by measuring the optical densities from immunofluorescence using FIJI software.

### In Situ Hybridization

The DIG‐labeled PLP probe was obtained as described (Chari et al., 2006), using mouse PLP cDNA (a kind gift from I.R. Griffiths, Glasgow, UK) that was cloned into the pGEM4 plasmid (Promega, Southampton, UK). To obtain the Fn probe, a cDNA fragment was generated by reverse transcription PCR, using total RNA isolated from WT mouse liver and the following Fn primers: forward: 5′‐CACCACCCAGAACTACGATG‐3′, reverse: 5′‐GGACACCATGCACAAACTTC‐3′. The cDNA was inserted into the pGEM4 plasmid (Promega, Southampton, UK). For both the PLP and Fn probes, antisense digoxygenin labeled cRNA probes (riboprobes) were then synthesized with appropriate RNA polymerase using a digoxigenin (DIG) RNA labeling kit (Roche, Lewes, UK). The size of the riboprobes was checked by agarose gel electrophoresis. *In situ* hybridization was then performed on air dried spinal cord sections as described previously (Chari et al., 2006).

### Real‐Time PCR

Total RNA was isolated from tissue homogenates using the RNeasy Mini kit (Qiagen). Reverse transcription of 0.5 µg total RNA was performed in the presence of oligo(dT)12–18 and dNTPs (Invitrogen) with SuperScript_II reverse transcriptase (Invitrogen) according to the manufacturer's instructions. Real‐time qPCR was performed using the Applied Biosystems 7900HT Real‐Time PCR System. Each reaction contained 5 ng cDNA, 10 pM primers (listed in Table 1) and ABsolute SYBR Green Rox mix (Thermo Scientific, Landsmeer, NL). No‐template controls were performed to ensure that amplification was not a result of contamination with genomic DNA. Gene expression levels were analyzed using the 2^−ΔΔct^ method (Livak, 2001), and relative expression to HPRT1 or GAPDH. Similar results were obtained for both housekeeping genes.

**Table 1:** Primer Sequences for Real‐Time qPCR

	Sense	Antisense
EIIIA‐fibronectin	GTTAGTGTCTATGCTCAGAACC	TCCACATCAGTGAATGCCAG
EIIIB‐fibronectin	AAAGATGACAAGGAAAGTGCCC	ATGGTGGAAGAGTTTAGCGG
Fibronectin	GTGAAAGGGAACCAGCAGAG	CTTGAAGCCAATCCTTGGAG
HPRT1	GACTTGCTCGAGATGTCA	TGTAATCCAGCAGGTCAG
GAPDH	CATCAAGAAGGTGGTGAAGC	ACCACCCTGTTGCTGTAG

### BrdU Incorporation Assay

OPCs were plated on 8‐well Permanox chamber‐slides (Nunc, Naperville, IL), precoated with 5 µg/mL PLL followed by appropriate aFn or pFn coatings (described above), at a density of 30,000 cells per well. OPCs were allowed to incorporate 5‐bromo‐2‐deoxyuridine (BrdU; 10 µM; Roche) for 24 h in the presence of 10 ng/mL PDGF‐AA and 10 ng/mL FGF‐2. Then, cells were fixed in 4% PFA for 20 min, and additionally fixed in 5% acetic acid in ethanol for 20 min. BrdU was detected using reagents from the BrdU labeling and Detection Kit I (Roche) according to the manufacturer's instructions with the addition of the oligodendrocyte lineage marker Olig2 (Abcam) and Alexa Fluor© 546‐conjugated anti‐rabbit antibody, and visualization of nuclei with DAPI (1 µg/mL). To compare the percentages of BrdU‐incorporating cells between the conditions, the numbers of double BrdU‐ and Olig2‐positive cells were counted relative to the Olig2‐positive cells (at least 150 cells per condition) from images captured with a Leica TCS SP8 Confocal Laser Scanning Microscope.

### Transwell Migration Assay

OPCs were seeded on pFn or aFn coated‐polyethylene terephthalate membranes of 8 µm pore size (Becton‐Dickinson Labware) in 12‐well modified Boyden transwell microchambers at a density of 80,000 cells per insert. OPCs were allowed to migrate through the membranes for 4 h using 10 ng/mL PDGF‐AA as a chemoattractant in the bottom of the well. Nonmigrating cells were removed from the top compartment with a cotton swab. Remaining cells in the membranes were fixed for 20 min in 5% acetic acid in ethanol and nuclei were visualized using DAPI (1 µg/mL) in PBS for 30 min. After washing in PBS, membranes were mounted on glass slides and images of DAPI‐positive, migrated cells were captured using a Zeiss Axioskop 2 plus microscope with Leica Application Suite v3 software (15 × 20 fields per membrane). The numbers of cells were assessed using FIJI software.

### Adhesion Assay

pFn or aFn coated 96‐wells plates (Nunc) were blocked for 30 min with 1% heat‐inactivated BSA at 37°C. Then, wells were left untreated or treated with the Fn blocking antibodies against EIIIA or EIIIB (mouse anti‐Fn IST9; Abcam and anti‐Fn C6; Abcam). After washing, 10,000 OPCs in 50 µL Sato medium per well were allowed to adhere for 1 h at 37°C. For integrin blocking experiments, OPCs were preincubated with anti‐integrin β1 (Becton Dickinson Pharmingen, Breda, NL; 1:200), anti‐integrin β3 (Becton Dickinson Pharmingen; 1:200) or anti‐integrin β5 (Millipore, Chemicon, Temecula, CA; 1:200) antibodies for 30 min at 37°C. The cells were washed two times with PBS, and adhered cells were fixed for 15 min with ice‐cold methanol. Cells were stained with 0.2% crystal violet (in 2% ethanol; Sigma), then washed several times with water, after which the stain was solubilized in 1% SDS. Adhesion was quantified by measuring the absorbance at 570 nm after 30 min. In each independent experiment, adhesion is expressed as percentage of the corresponding untreated substrate control resulting from triplicate determinations.

### Statistical Analyses

Statistical analyses were performed in GraphPad Prism (GraphPad, La Jolle, CA). First, the Kolmogorov‐Smirnov test was applied to test for a normal distribution of the data. Multiple group comparisons of data, which could thus be assumed to have a normal distribution (data sets of Fn^cKO^ mice, derived from immunohistochemistry and *in situ* hybridization experiments, except for the data describing immunofluorescence intensity of Fn‐ and Iba1‐staining), were performed using one‐way ANOVA followed by Tukey's Multiple Comparison Test (* *P* < 0.05; ** *P* < 0.01; *** *P* < 0.001). Multiple group comparisons of data that were not compatible with a normal distribution (data sets derived from *in vitro* assays and real‐time qPCR on demyelinated tissue, as well as of the data describing immunofluorescence intensity of Fn and Iba1 stainings) were performed using the Kruskal‐Wallis test followed by Dunn's Multiple Comparison Test (*P < 0.05; ** *P* < 0.01; *** *P* < 0.001). Nonparametric ranking data, derived from blindly ranking alkaline toluidine blue stained resin sections for estimated degrees of remyelination, were statistically analyzed using the Mann‐Whitney test (Ibanez et al., 2004). For the *in vivo* mice data from immunohistochemistry, *in situ* hybridization and real time qPCR studies, a representative graph of 2–3 independent experiments is shown, displaying absolute means of 3–4 mice per group and 3 lesions levels per animal, each ∼120 µm from each other, with the exception of immunochemistry for Iba1, data of which are shown as a relative to the mean immunofluorescence levels of littermate WT controls, set at 100% for each independent experiment. Quantification methods are described under “immunochemistry.” For *in vitro* proliferation, migration, and adhesion assays, graphs display mean values relative to a control condition (aFn or pFn) from 3 to 5 independent experiments. All error bars represent standard deviations.

## Results

### Fibronectin in Demyelinated Lesions is Predominantly Produced by Astrocytes

Transient Fn expression during demyelination is hypothesized to benefit remyelination (Hibbits et al., 2012; Stoffels et al., 2013a; Zhao et al., 2009). The aim of the present study was therefore to better define the potential involvement and underlying mechanism(s) of the different Fn sources in remyelination. We used a well‐established rodent model of experimental demyelination, in which focal, primary demyelination is created by the injection of lysolecithin into the white matter of the spinal cord ventral funiculus (Blakemore and Franklin, 2008). Spontaneous and complete remyelination of these lesions proceeds over a period of ∼21–28 days in young adult rodents, which involves proliferation and migration of local, activated OPCs to the demyelinated lesions [“recruitment”; 1–10 days post lesion (DPL)], followed by differentiation of oligodendrocytes and myelin sheath formation (10–21 DPL) (Zhao et al., 2006). To eliminate pFn and cellular Fn from lysolecithin‐demyelinated lesions, we used Cre/lox technology, creating conditional knockout adult mice devoid of plasma Fn (pFn^cKO^), astrocyte Fn (aFn^cKO^) or both (a + pFn^cKO^). The pFn null allele was generated by activation of the Mx‐Cre promoter with poly I:C as described (Sakai et al., 2001), which removes the start codon, signal sequence and the exon/intron border of exon 1 from the pFn gene. To remove cellular Fn, we targeted astrocytes, because they are considered a major source of Fn in the CNS following injury (Hibbits et al., 2012), and synthesize pathological Fn aggregates in MS (Stoffels et al., 2013a). We thus used mice expressing Cre recombinase under control of a GFAP promoter, which was rendered active by exposure to tamoxifen via a modified estrogen receptor. The induction efficiency was tested in a reporter line, as described in a previous report (Hirrlinger et al., 2006). In our own experiments, this approach showed that at 5 days after a course of tamoxifen administration in unlesioned adult mice, of astrocytes in spinal cord white matter on average 78.5% ± 2.4% SD (*n* = 4) expressed the reporter (yellow fluorescent protein), indicating an efficient recombination.

**Figure 1:**
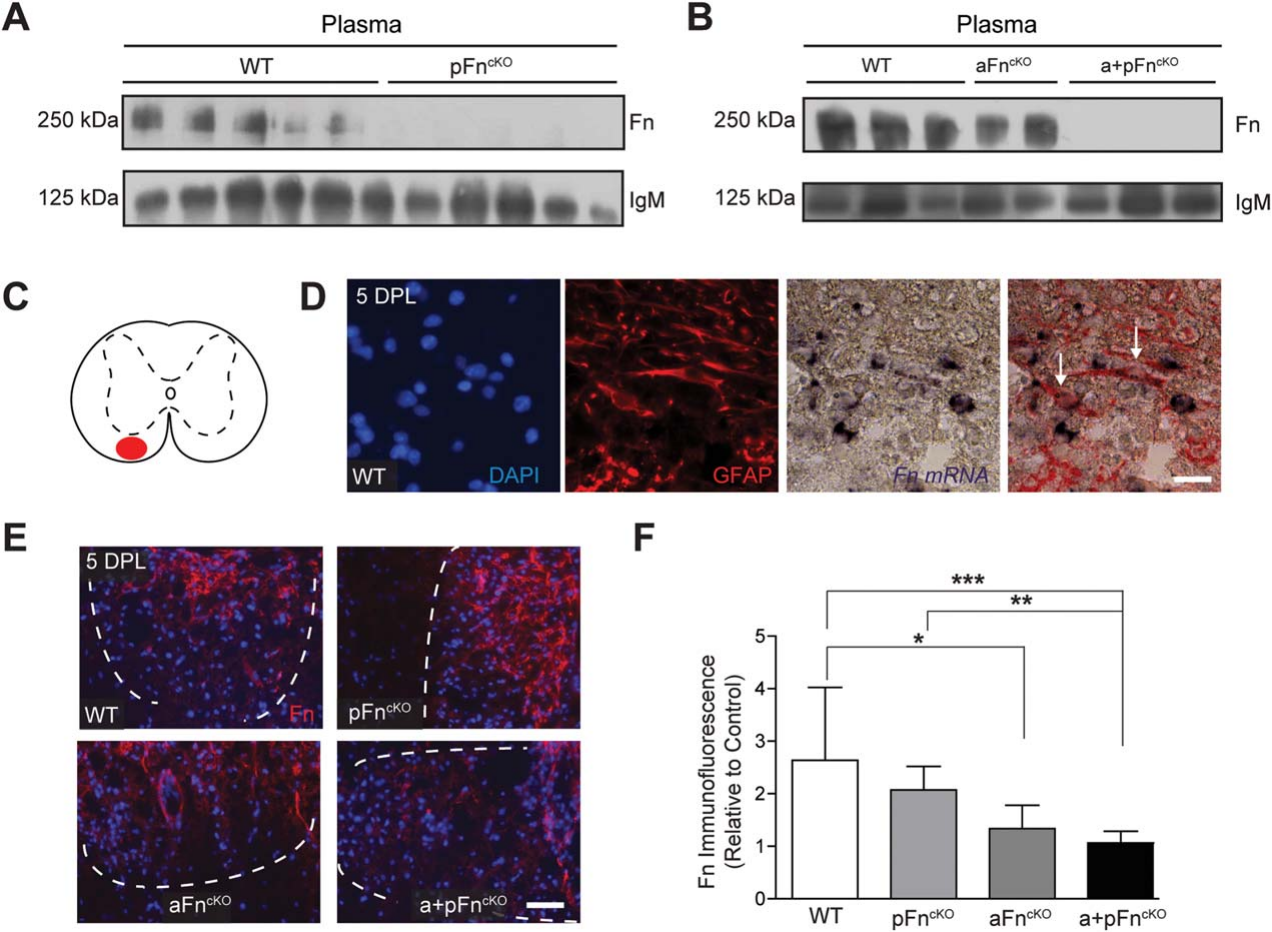
Fibronectin expression is decreased in plasma and demyelinated white matter of plasma, astrocyte, and astrocyte + plasma fibronectin conditional knockout mice. **A** and **B**: Western blot analysis for fibronectin (Fn) and immunoglobulin M (IgM) on plasma of littermate wild type (WT) mice and after conditional knockout of Fn from plasma (pFn^cKO^), astrocytes (aFn^cKO^) or astrocytes, and plasma (a + pFn^cKO^). Note the virtual absence of Fn from plasma after pFn^cKO^ and a + pFn^cKO^. **C**: Demyelination was induced by injection of lysolecithin into the white matter of the spinal cord ventral funiculus (red area). **D**: Immunohistochemistry for DAPI (cell nuclei) and GFAP (astrocytes) combined with *in situ* hybridization for Fn mRNA on lysolecithin‐demyelinated lesions of WT mice at 5 DPL, showing that Fn mRNA is present in astrocytes. **E**: Immunohistochemistry for Fn on lysolecithin‐demyelinated lesions at 5 DPL after pFn^cKO^, aFn^cKO^, a + pFn^cKO^, or in WT mice. F: Quantification of immunofluorescent staining intensities in (E) from 3 mice per group, and 3 lesion levels per animal, ∼120 μm distant from each other from images that were taken on the same day using the exact same camera settings. Note the reduction in intensity of immunofluorescence relative to WT after aFn^cKO^ and a + pFn^cKO^, but not following pFn^cKO^. Bars represent means. Error bars show standard deviations. Statistical analysis was performed using the Kruskal‐Wallis test followed by Dunn's Multiple Comparison Test (* *P* < 0.05; ** *P* < 0.01; *** *P* < 0.001). Demyelinated lesion outlines were measured in Zeiss Axovision 4.8 software based on the increase in DAPI staining inside the lesions, and outlines are represented by dashed white lines. Scale bar is 20 μm (D) and 50 μm (E). [Color figure can be viewed in the online issue, which is available at wileyonlinelibrary.com.]

Using this approach, pFn was successfully eliminated from plasma in pFn^cKO^ mice, as revealed by Western blot analysis (Fig. 1A). Similar results were obtained on plasma from a + pFn^cKO^ mice, whereas pFn remained present in the plasma of aFn^cKO^ mice as expected (Fig. 1B). Given the virtual absence of aFn from the healthy CNS and its increased expression after demyelination (Stoffels et al., 2013a; Zhao et al., 2009), we assessed local Fn expression in aFn^cKO^ and a + pFn^cKO^ mice after lysolecithin‐induced demyelination of the spinal cord ventral funiculus white matter (Fig. 1C). First, we confirmed that astrocytes are a source of cellular Fn in mouse demyelinated lesions, using immunhistochemistry against GFAP and *in situ* hybridization for Fn mRNA on WT lesions (Fig. 1D). Further, as shown in Fig. 1E,F, Fn expression was increased in demyelinated lesions of littermate WT mice at 5 DPL, as previously reported for rat models (Stoffels et al., 2013a; Zhao et al., 2009). Moreover, this increase was transient, and Fn levels were decreased in mice at 14 DPL (cf Fig. 5, data not shown), similar to the transient expression pattern of Fn observed in rats (Stoffels et al., 2013a; Zhao et al., 2009). In pFn^cKO^ mice, Fn immunostaining was unaltered compared with expression in littermate WT mice (Fig. 1E,F), despite the strong reduction in Fn plasma levels (Fig. 1A). These data indicate that either pFn is not a major source of Fn expression within lysolecithin‐induced lesions, or that aFn expression may compensate for the loss of pFn. In contrast, Fn expression was markedly reduced in demyelinated lesions of aFn^cKO^ and a + pFn^cKO^ mice (Fig. 1E,F), corroborating that astrocytes are a major source of cellular Fn after demyelination. Having reduced Fn levels from plasma and astrocytes, we next analyzed how remyelination was affected at both the recruitment and differentiation stages of remyelination.

### Proliferation of OPCs in Response to Demyelination is Decreased in Astrocyte Fibronectin Conditional Knockout Mice

**Figure 2:**
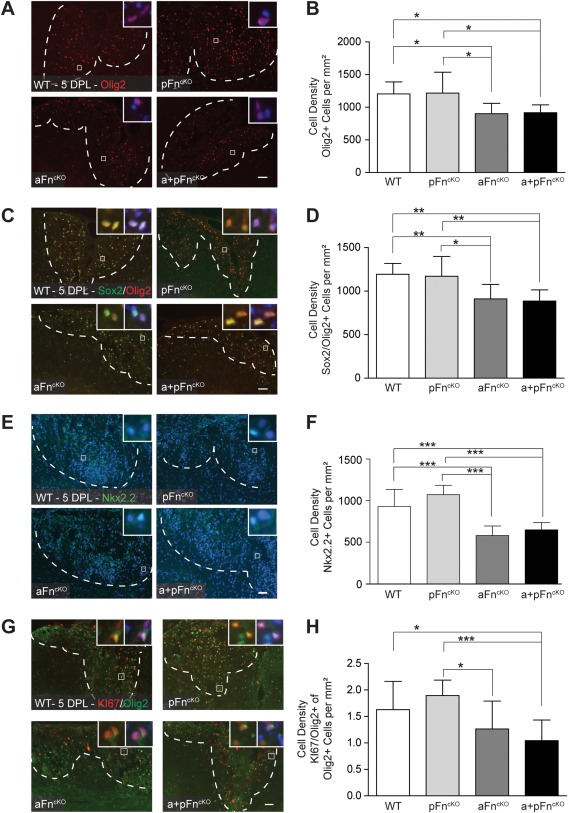
OPC numbers in lysolecithin‐induced demyelinated lesions decrease after conditional knockout of astrocyte fibronectin, but not after plasma fibronectin conditional knockout alone. **A–I**: After 5‐days post lysolecithin‐demyelinated lesions (5 DPL) of the mouse spinal cord ventral funiculus, the density of Olig2‐positive (“Olig2+”; A,B), Sox2+ Olig2+ (C,D), Nkx2.2+ (E,F), and KI67+Olig2+ (G,H) cells were determined by immunohistochemistry. Note the decrease in Olig2+, Sox2+Olig2+, Nkx2.2+, and KI67+Olig2+ cells number after conditional knockout of fibronectin from astrocytes (aFn^cKO^) or from astrocytes and plasma (a + pFn^cKO^), but not from plasma alone (pFn^cKO^) relative to littermate WT mice. Images in A,C,E and G are representative images of demyelinated areas in the different groups. Insets show higher power magnifications of the double positive cells that were counted, with the blue color representing DAPI staining. The outline of the demyelinated lesions was measured in Zeiss Axovision 4.8 software based on increased DAPI stainings' inside lesions and outlines are represented by dashed white lines. Double (C,G) or single (A,E) positive cell numbers were manually counted 3 times for 4 mice per group, and 3 lesion levels per animal, ∼120 μm distant from each other. Representative graphs of 2–3 independent experiments are shown. Bars represent means. Error bars show standard deviations. Statistical analyses were performed using one‐way ANOVA, followed by Tukey's Multiple Comparison Test (* *P* < 0.05; ** *P* < 0.01; *** *P* < 0.001; NS: not significant). Scale bars are 50 μm. [Color figure can be viewed in the online issue, which is available at wileyonlinelibrary.com.]

**Figure 3:**
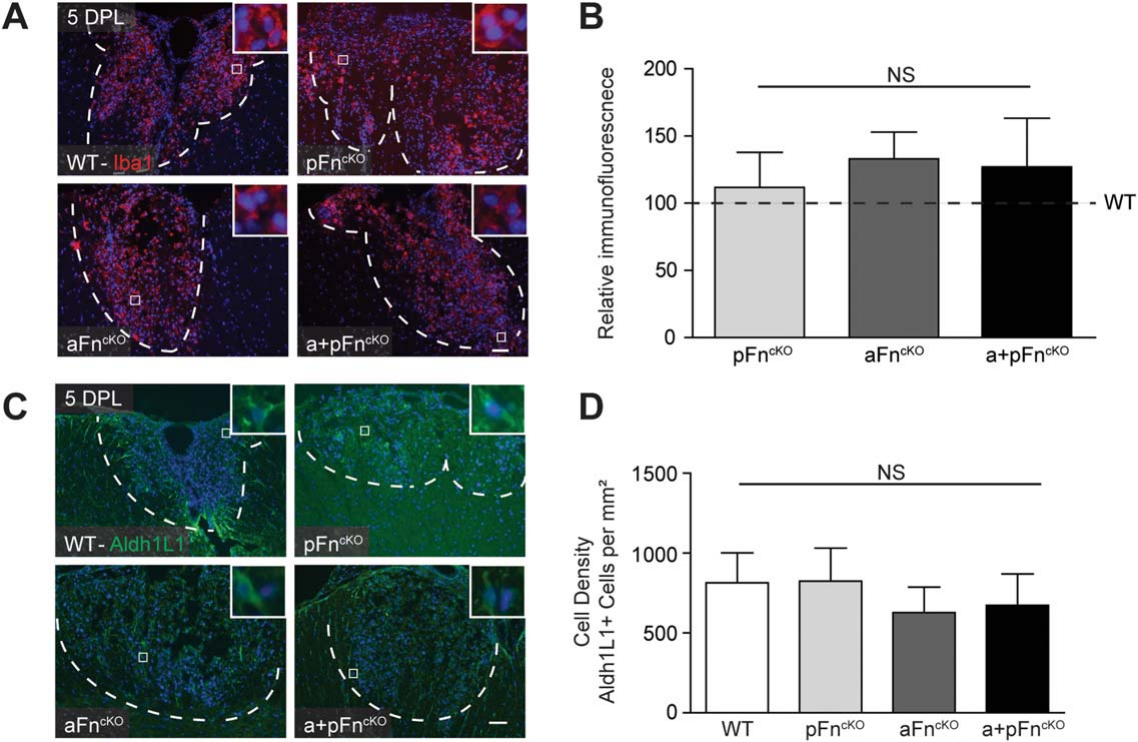
Microglia and astrocyte cell numbers in lysolecithin‐demyelinated lesions are not affected by conditional knockout of plasma and/or astrocyte fibronectin. **A–D**: After 5 DPL of the mouse spinal cord ventral funiculus from littermate WT mice and after conditional knockout of Fn from plasma (pFn^cKO^), astrocytes (aFn^cKO^), or astrocytes and plasma (a + pFn^cKO^)., the density of Iba1+ cells was determined by measurement of relative immunofluorescence (A,B), and Aldh1L1+ cells were stained by immunohistochemistry, then counted (C,D). Representative images of demyelinated areas in the different groups are shown. Insets show higher power magnifications of the double positive cells, with the blue color representing DAPI staining. The outline of the demyelinated lesions was measured in Zeiss Axovision 4.8 software based on increased DAPI stainings' inside lesions and outlines are represented by dashed white lines. Immunofluorescence levels of Iba+ cells were measured using FIJI software (A,B) and Aldh1L1+ cell numbers were manually counted 3 times for 4 mice per group, and 3 lesion levels per animal, ∼120 μm distant from each other. Representative graphs of 2–3 independent experiments are shown. Bars represent means. Error bars show standard deviations. Statistical analyses were performed using the Kruskal‐Wallis test followed by Dunn's Multiple Comparison Test (A,B) or one‐way ANOVA, followed by Tukey's Multiple Comparison Test (C,D) (*P < 0.05; ** *P* < 0.01; *** *P* < 0.001; NS: not significant). Scale bars are 50 μm. [Color figure can be viewed in the online issue, which is available at wileyonlinelibrary.com.]

To determine whether the density of OPCs in the demyelinated area is affected by pFn^cKO^ and/or aFn^cKO^, we analyzed OPC numbers after lysolecithin‐induced demyelination in the different Fn^cKO^ mice. Immunostaining for Olig2 was used as a marker for oligodendrocyte lineage cells (Arnett et al., 2004; Fancy et al., 2009) and Sox2 and Olig2 double immunohistochemistry to identify OPCs (Kondo and Raff, 2004; Shen et al., 2008; Fig. 2A–D). This approach revealed a small, significant reduction in Olig2‐positive (Olig2+) cells and Sox2+Olig2+ cells after aFn^cKO^, but not pFn^cKO^ at 5 DPL (Fig. 2A–D). A similar reduction in Olig2+ and Sox2+Olig2+ cells was detected after a+pFn^cKO^ (Fig. 2A–D), indicating that the decrease in OPC density was associated with the decrease in aFn. An additional decrease in pFn levels in a + pFn^cKO^ did not further reduce the number of OPCs. In support of these observations, by determining OPC density using the transcription factor Nkx2.2 as a marker (Watanabe et al., 2004), we similarly detected that the OPC density was reduced after aFn^cKO^ and a + pFn^cKO^ (Fig. 2E,F). The lesions were of comparable sizes in the different Fn^cKO^ samples relative to WT (data not shown). In tissue culture, pFn promotes OPC migration and proliferation (Baron et al., 2002; Hu et al., 2009; Milner et al., 1996). To examine whether the reduction in OPC numbers in aFn^cKO^ mice reflected an impairment in proliferation, we next analyzed the numbers of proliferating OPCs at 5 DPL, determined by co‐labeling with Olig2 and the cell proliferation marker KI67 (Gerdes et al., 1983). This revealed a reduction in KI67+Olig2+ cells relative to Olig2+ cells in aFn^cKO^ and a + pFn^cKO^, but not pFn^cKO^ (Fig. 2G,H), indicating that the decrease in OPC numbers is, at least partly, a result of impaired proliferation. In contrast, we did not observe a substantial change in microglia/macrophage numbers after Fn^cKO^, as assessed by immunohistochemistry for ionized calcium‐binding adaptor molecule 1 (Iba1) (Imai et al., 1996; Fig. 3A,B). Similarly, astrocyte numbers were not significantly affected by Fn^cKO^, as reflected by comparable numbers of cells that stained positive for aldehyde dehydrogenase 1, member L1 (Aldh1L1; Cahoy et al., 2008; Lovatt et al., 2007; Fig. 3C,D). Hence, aFn^cKO^ resulted in a reduced number of OPCs after demyelination, whereas microglia/macrophage and astrocyte cell numbers were unaffected.

### Oligodendrocyte Differentiation and Remyelination are not Affected by Conditional Knockout of Fibronectin from Astrocytes

**Figure 4:**
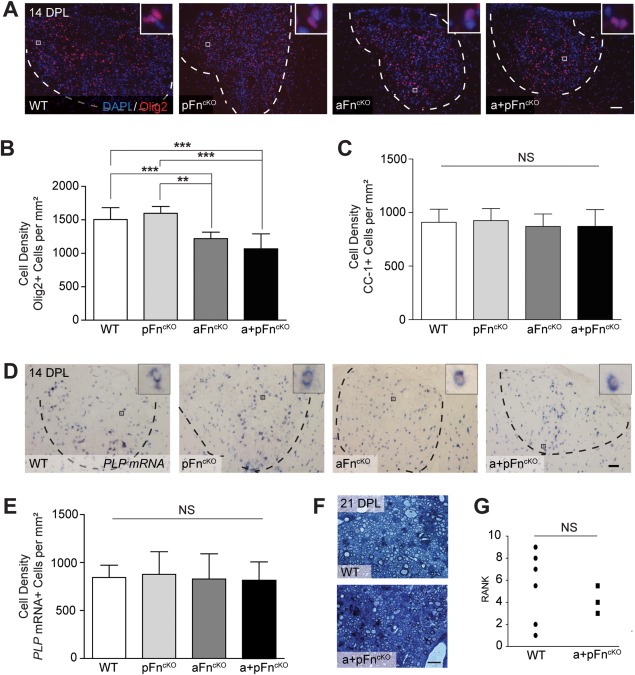
Oligodendrocyte differentiation and remyelination are not affected by conditional knockout of fibronectin from astrocytes. **A–E**: At 14 days post lysolecithin‐induced demyelination (14 DPL) of the spinal cord ventral funiculus, immunohistochemistry (A–C) and *in situ* hybridization (D,E) were applied to determine the numbers of Olig2+ (A,B), CC1+ (C), and PLP mRNA+ (D,E) cells in littermate WT mice or after conditional knockout of fibronectin from plasma (pFn^cKO^), astrocytes (aFn^cKO^), or astrocytes, and plasma (a + pFn^cKO^). Note the significant decrease of Olig2+ oligodendrocyte lineage cells after aFn^cKO^ and a + pFn^cKO^ (A,B), whereas the numbers of differentiating oligodendrocytes do not differ from littermate WT mice (C–E). Images in A and D are representative images of demyelinated areas in the different groups. Insets show higher power magnifications of the positive cells that were counted, with the blue color representing DAPI staining. Outlines of demyelinated lesions were measured in Zeiss Axovision 4.8 software based on the increase in DAPI staining inside lesions, and outlines are represented by dashed lines. Cell numbers were manually counted 3 times from 4 mice per group, and 3 lesion levels per animal, ∼120 μm distant from each other. Representative graphs of 2–3 independent experiments are shown. Bars represent means of each group. Error bars show standard deviations. Statistical analyses were performed using one‐way ANOVA, followed by Tukey's Multiple Comparison Test (** *P* < 0.01; *** *P* < 0.001; NS: not significant). Scale bars are 50 μm. F,G. At 21 DPL, semi‐thin sections of resin‐embedded WT and a + pFn^cKO^ mice were stained with alkaline toluidin blue to analyze the myelin structure (**F**), and blindly ranked according to estimated percentage of remyelination by two independent researchers, after which statistical differences were analyzed using the Mann Whitney test (G). Scale bar is 100 μm. NS means “not significant.” [Color figure can be viewed in the online issue, which is available at wileyonlinelibrary.com.]

We next examined whether the decreased OPC density observed in aFn^cKO^ and a + pFn^cKO^ during demyelination resulted in altered CNS remyelination following lysolecithin‐induced demyelination. Oligodendrocyte lineage cell numbers were analyzed by immunohistochemistry for Olig2 at 14 days post lysolecithin‐induced demyelination. As shown in Fig. 4A,B, Olig2+ cells were still decreased in a + pFn^cKO^ and aFn^cKO^ as compared with littermate WT mice. In pFn^cKO^ mice, Olig2+ cell numbers did not differ from the WT mice in the lesions. Furthermore, analysis of differentiated oligodendrocytes, determined by immunohistochemical staining for CC1 (Bhat et al., 1996), showed that the density of CC1+ cells was similar in all groups (Fig. 4C). This was confirmed using an alternative marker of mature oligodendrocytes, *in situ* hybridization for proteolipid protein (PLP) mRNA, a major myelin protein, which revealed no differences in PLP mRNA+ cells (Fig. 4D,E). Therefore, despite a reduction of OPCs after demyelination in aFn^cKO^ and a + pFn^cKO^, it was not sufficient to result in an impairment of oligodendrocyte generation at 14 DPL.

**Figure 5:**
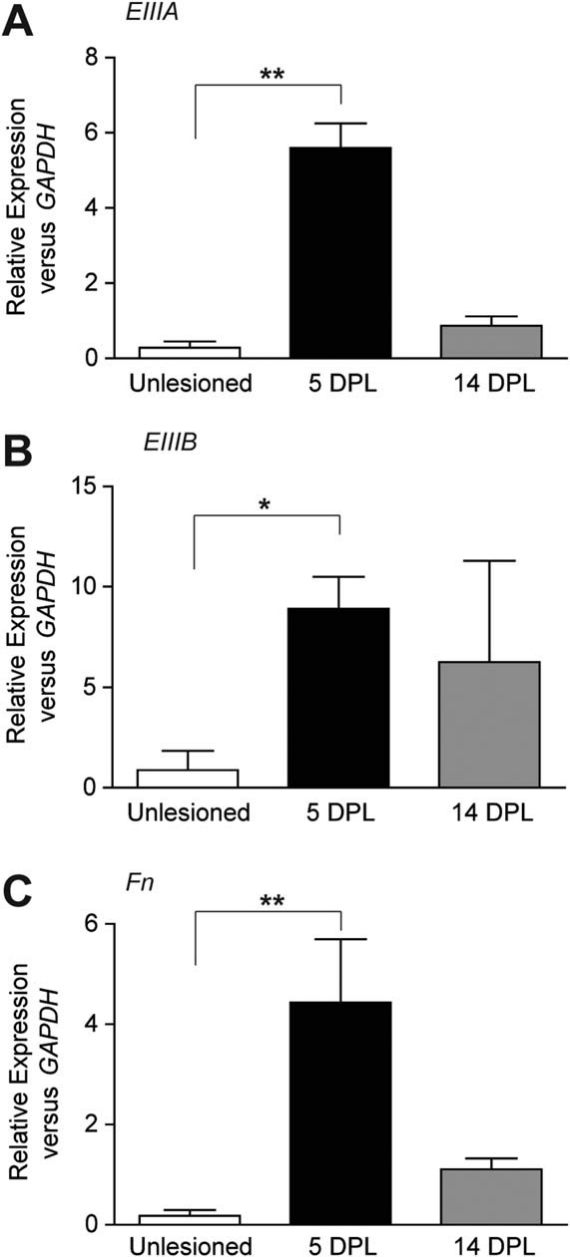
Enhanced expression of fibronectin alternatively spliced domains EIIIA and EIIIB mRNA after lysolecithin‐induced demyelination of the mouse spinal cord ventral funiculus. **A–C**: Real‐time PCR on spinal cord RNA from control mice (unlesioned), at 5 days (5 DPL) or 14 days (14 DPL) post lysolecithin‐induced demyelination. Note the increase in fibronectin EIIIA and EIIIB mRNA at 5 DPL. Gene expression levels were analyzed using the 2^−ΔΔct^ method (Livak, 2001), with relative expression to HPRT1 or GAPDH. Similar results were obtained for both housekeeping genes; graphs shown the relative expression to GAPDH. Statistical analysis was performed using the Kruskal‐Wallis Test, followed by Dunn's Multiple Comparison Test (* *P* < 0.05; ** < *P* 0.01).

To assess whether myelin sheath formation was affected, we examined toluidine blue stained semithin resin sections of lesions from littermate WT and a + pFn^cKO^ mice at 21 DPL. We could not detect any gross morphological differences between remyelinated lesions from littermate WT and a + pFn^cKO^ mice (Fig. 4F). Ranking analysis of the degree of remyelination (Ibanez et al., 2004) did not reveal significant differences between WT and a + pFn^cKO^ lesions either (Fig. 4G). Therefore, aFn, although involved in OPC recruitment, is not essential for remyelination.

### Expression of the Alternatively Spliced Domains EIIIA and EIIIB mRNA of Cellular Fibronectin is Increased Following Demyelination

We next investigated the specific contribution of the alternatively spliced domains EIIIA and EIIIB, exclusively localized within the cellular Fn structure (Paul et al., 1986; Schwarzbauer et al., 1987) in the regulation of OPC proliferation following demyelination. We first determined the expression of both domains following demyelination of the mouse spinal cord ventral funiculus by quantitative, real‐time PCR analysis of mRNA expression. Compared with unlesioned tissue, EIIIA, EIIIB, and total Fn mRNA levels were increased in demyelinated tissue at 5 DPL relative to expression of the housekeeping gene GAPDH (Fig. 5A–C; 5 DPL), similar to our previous report for total Fn protein and Fn EIIIA protein (Stoffels et al., 2013a). Furthermore, during remyelination (14 DPL), mRNA levels of EIIIA, EIIIB, and total Fn returned to lower levels (Fig. 5A–C; 14 DPL). Because both EIIIA and EIIIB mRNA levels are increased at 5 DPL, when OPCs are recruited, and since these elements are exclusively present in Fn derived from local cellular sources, such as astrocytes, we next examined the extent to which aFn EIIIA, EIIIB, or both are involved in proliferation and/or migration of OPCs *in vitro*.

### The EIIIA Domain of Astrocyte‐Derived Fibronectin Mediates Proliferation of OPCs, but not Their Migration and Adhesion

**Figure 6:**
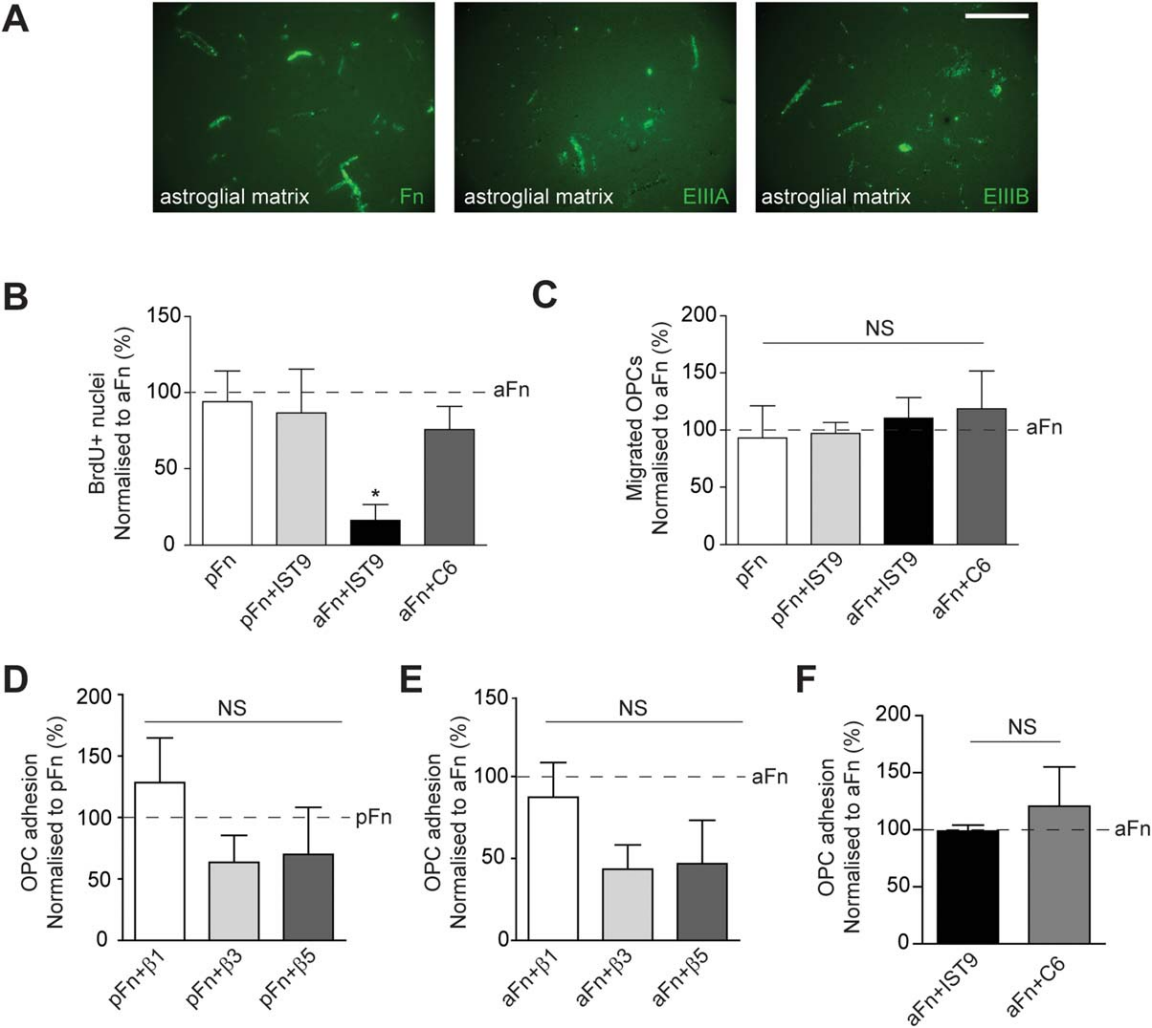
Blocking the EIIIA domain from astrocyte‐derived fibronectin affects OPC proliferation, but not migration and adhesion. **A**: Astroglial matrix derived from neonatal rat astrocytes (aFn) immunostained for total fibronectin (“Fn”), and the alternatively spliced EIIIA (“EIIIA”) or EIIIB (“EIIIB”) domains. Scale bar is 50 μM. **B**: OPCs from neonatal rats were cultured for 24 h on plasma fibronectin (pFn) or aFn, alone or in the presence of blocking antibodies against the EIIIA or EIIIB domain and subjected to a BrdU incorporation assay combined with Olig2 immuncytochemistry. On aFn, 16% of OPCs proliferated on average ± 5% SD. Note that addition of an EIIIA blocking antibody (“aFn + IST9”) decreased the number of Olig2+ OPCs incorporating BrdU on aFn, but not on pFn (“pFn + IST9”), and BrdU incorporation did not differ between the other conditions. **C**: OPCs were allowed to migrate through transwell inserts coated with aFn or pFn for 4 h, alone or in the presence of blocking antibodies against EIIIA (“pFn + IST9”; “aFn + IST9”) or EIIIB (“aFn + C6”) antibodies. Note that the numbers of migrated cells did not differ between the conditions. **D–F**: OPCs were allowed to adhere to pFn (D) or aFn (E,F) in the presence of blocking antibodies against integrins β1 (“pFn + β1,” “aFn + β1”), β3 (“pFn + β3,” “aFn + β3”), or β5 (“pFn + β5”, “aFn + β5”; D,E) or after blocking the EIIIA (“aFn + IST9”) or EIIIB (“aFn + C6”; F) domains of aFn. Note that whereas integrins β3 and β5 likely mediate adhesion of OPCs, particularly to aFn (E), adhesion does not require a functional EIIIA or EIIIB domain on aFn (F). Bars in the graphs represent means of 3–4 independent experiments. The horizontal, dashed line represents pFn (D) or aFn‐treated (B, C, E, F) cells, and this condition was set to 100% in each experiment. Error bars show standard deviations. Statistical analyses were performed using the Kruskal‐Wallis Test, followed by Dunn's Multiple Comparison Test (** *P* < 0.01; NS: not significant). [Color figure can be viewed in the online issue, which is available at wileyonlinelibrary.com.]

To examine the effect of pFn and aFn on OPC proliferation, we used commercially available pFn from bovine plasma and aFn derived from extracellular deposits of primary astrocytes (Fig. 6A, “Fn”). The aFn preparation contained the alternatively spliced domain EIIIA (Fig. 6A, “EIIIA”) and EIIIB (Fig. 6A, “EIIIB”), as confirmed by immunostaining with domain‐specific antibodies. Functional blocking antibodies against EIIIA (“IST9”, Liao et al., 1999) and EIIIB (“C6”, Balza et al., 2009) were used to eliminate signals from these domains. OPCs were allowed to proliferate on the different substrates in the presence of relatively high levels of PDGF‐AA and FGF‐2 for 24 h, after which percentages of BrdU‐incorporating Olig2+ cells were determined by immunocytochemistry as a measure of OPC proliferation. As expected under these conditions (Baron et al., 2002, Colognato et al., 2002), OPCs proliferated equally well on either aFn (16 ± 5% SD) or pFn (15 ± 8% SD; Fig. 6B), and to comparable levels when grown on PLL (data not shown). However, proliferation of OPCs was markedly reduced when the cells were cultured on aFn with the EIIIA domain blocked (Fig. 6B; “aFn + IST9”). In contrast, addition of the EIIIA blocking antibody to pFn did not affect OPC proliferation (Fig. 6B; “pFn + IST9”), indicating that the reduction in OPC proliferation following the blocking of EIIIA with the IST9 antibody was attributable to the specific effect(s) of the antibody. Moreover, blocking EIIIB from aFn (Fig. 6A; “EIIIB”) with the C6 antibody (Fig. 6B; “aFn + C6”) did not affect OPC proliferation. To determine whether EIIIA and/or EIIIB of aFn are also important for OPC migration, we allowed OPCs to migrate through transwell microchambers, containing membranes coated with pFn or aFn on both sides, using PDGF‐AA as an attractant in the bottom well. As shown in Fig. 6C, migration of OPCs was similar on aFn and pFn, and was not affected by blocking the EIIIA or EIIIB domains. These experiments demonstrate that EIIIA, but not EIIIB, is primarily required for proliferation of OPCs on aFn, and that neither domain is essential for migration of OPCs on aFn.

Proliferation of OPCs on pFn at low levels of PDGF‐AA is largely mediated by the integrin receptors αvβ3 and to a lesser extent by αvβ1 (Baron et al., 2002; Blaschuk et al., 2000; Milner et al., 1996). Although EIIIA does not directly bind to αvβ1 or αvβ3, it has been reported that EIIIA may indirectly promote adhesion of cellular Fn to these integrins, thereby facilitating proliferation (Manabe et al., 1999). To test this possibility, we assessed whether adhesion of OPCs to aFn indirectly involved integrin β binding by using blocking antibodies against integrins β1, β3 and β5, and whether such adhesion was facilitated by functional EIIIA and EIIIB domains. Compared with adhesion to pFn (Fig. 6D; “pFn + β3” and “pFn + β5”), adhesion of OPCs to aFn was only modestly reduced when the integrins β3 (Fig. 6E; “aFn + β3”) and β5 (Fig. 6E; “aFn + β5”) were blocked. In contrast, integrin β1 had no effect on adhesion of OPCs to both pFn and aFn (Fig. 6D,E; “pFn + β1” and “aFn + β1”). Furthermore, functional blocking of EIIIA and EIIIB domains did not alter adhesion of OPCs to aFn (Fig. 6F). Thus, whereas the integrin β3 and β5 receptors likely mediate adhesion of OPCs to aFn, adhesion of OPCs does not require functional EIIIA or EIIIB domains of aFn.

## Discussion

In this study, we investigated how Fn, derived from plasma (pFn) or cellular Fn derived from astrocytes (aFn), modulates remyelination following CNS demyelination. After conditional knockout of Fn from astrocytes (aFn^cKO^) or both astrocytes and plasma (a + pFn^cKO^), the density of (proliferating) OPCs in demyelinated lesions was reduced. Our *in vitro* analyses of OPCs, cultured on either pFn or aFn, revealed that the alternatively spliced EIIIA domain, exclusively present in cellular Fn, is instrumental in proliferation. Our data further showed that this control of proliferation by the EIIIA domain was likely not related to an ability of EIIIA to enhance adhesion of OPCs, although integrins β3 and β5 mediate adhesion of OPCs to aFn. Furthermore, we did not observe an effect of aFn on migration of OPCs. Despite a reduction in oligodendrocyte lineage cells on a + pFn^cKO^ at later stages of the remyelination process, both pFn and aFn were dispensable for oligodendrocyte differentiation and complete remyelination.

Although both EIIIA and EIIIB are largely absent from healthy tissue after embryonic development, reappearance of cellular Fn with the alternatively spliced EIIIA and EIIIB domains occurs after injury of different tissue types (ffrench‐Constant et al., 1989; Jarnagin et al., 1994; Nickeleit et al., 1996; Ulrich et al., 1997). Injury‐induced expression of cellular Fn containing EIIIA is associated with increased cell proliferation, as demonstrated in a variety of cell types (Manabe et al., 1999; Olsen et al., 2012; Stenzel et al., 2011). In addition, while proliferation of OPCs is known to be independent of pFn at sufficient levels of mitogenic growth factors (Baron et al., 2002, Colognato et al., 2004), which are likely also sufficient in lysolecithin‐induced demyelinated lesions (Hinks and Franklin, 1999), our data indicate that the EIIIA domain is essential for proliferation of OPCs on aFn under these conditions. This suggests that proliferation of OPCs on aFn involves a different mechanism from pFn. The EIIIA could theoretically mediate proliferation of OPCs on aFn via binding to the integrins α9β1 (Olsen et al., 2012; Ou et al., 2013; Sun et al., 2014) and α4β1 (Liao et al., 2002), or by enhancing the binding affinity of other integrin receptors for Fn, most notably α5β1 and αvβ3 (Manabe et al., 1997, 1999; Xia and Culp, 1995). However, integrins α4β1 and α9β1 are not expressed by OPCs (Milner and ffrench‐Constant, 1994) and the EIIIA domain was not important for adhesion of OPCs to aFn, implying that EIIIA mediates proliferation of OPCs through an alternative mechanism. This mechanism may involve other domains of Fn, since alternative splicing of Fn can change the conformation of Fn, affecting the presentation of binding sequences and exposing cryptic binding sites (Pickford and Campbell, 2004; Ventura et al., 2010). In addition to EIIIA, expression of the EIIIB domain was also upregulated after demyelination. Whereas EIIIB appears not to have a role in proliferation and migration of OPCs, its functional involvement in processes other than proliferation and migration cannot be excluded and merits further investigation.

The apparent redundancy of pFn for achieving a normal cell density of OPCs observed in our *in vivo* studies was unexpected, given that stimulation of cell proliferation and migration by pFn is well documented for several cell types (To and Midwood, 2011; von Au et al., 2013), including OPCs at low growth factor levels (Baron et al., 2002; Colognato et al., 2004; Hu et al., 2009; Milner et al., 1996). Our *in vitro* proliferation studies suggested that OPCs proliferate equally on pFn and aFn in the presence of PDGF‐AA and FGF‐2. After pFn^cKO^, a compensatory increase in cellular Fn may have occurred, mediated by astrocytes, microglia/macrophages, and endothelial cells (Stoffels et al., 2013a). From these cell types, astrocytes are thought to represent the major source of Fn after toxin‐induced demyelination (Hibbits et al., 2012; Stoffels et al., 2013a), which is further supported in this study, given the pronounced reduction in Fn levels after aFn^cKO^. Therefore, a compensatory increase in aFn could explain the absence of a clear phenotype after pFn^cKO^, in agreement with studies of other tissues (Sakai et al., 2001). However, the observation that a + pFn^cKO^ does not substantially amplify the reduction in OPC numbers during demyelination may support an alternative explanation, namely that excessive leakage of pFn to the demyelinated area does not occur on lysolecithin‐induced demyelination, despite breakdown of the blood‐brain barrier (Ford et al., 1990). Hence, in contrast to previous studies, in which pFn was considered a predominant source of the Fn matrix in tissue (Moretti et al., 2007), our findings indicate that aFn rather than pFn is the major component of the Fn matrix expressed after lysolecithin‐induced CNS demyelination. In MS lesions, where immune‐mediated blood‐brain barrier disruption is more diffuse, pFn may be a more prominent source of Fn. Also, pFn could potentially modulate OPC proliferation when growth factor levels are low (Baron et al., 2002; Colognato et al., 2004).

Although aFn mediates OPC cell numbers in demyelinated lesions, it is dispensable for remyelination. Moreover, at the stage of oligodendrocyte differentiation, the absolute numbers of differentiated oligodendrocytes did not differ between aFn^cKO^ or a + pFn^cKO^ and WT animals. After lysolecithin‐induced demyelination in WT animals, the amount of differentiating oligodendrocytes remains relatively stable from this stage onwards until the completion of remyelination (Fancy et al., 2004; our unpublished observations). This indicates that despite the reduced numbers of OPCs after aFn^cKO^ or a + pFn^cKO^, sufficient numbers of differentiated oligodendrocytes are still generated to reach the maximal level of differentiation necessary to remyelinate the lesion. In this way, relative redundancy of aFn, in spite of decreased OPC proliferation after aFn^cKO^, is consistent with the concept that OPCs are normally recruited in excess relative to the numbers required for remyelination following small, focal, toxin‐induced lesions (Franklin and ffrench‐Constant, 2008; Stidworthy et al., 2004). In addition, functional compensation may result from the increased expression of several other ECM proteins in demyelinated lesions (Zhao et al., 2009). In support of this, osteopontin, another ECM molecule expressed after demyelination, is also redundant for remyelination (Zhao et al., 2008). In contrast to modulation of OPC proliferation by aFn, which likely promotes recovery, persistent expression of Fn variants in pathology mediates failure of tissue regeneration (Stoffels et al., 2013b). In particular, the EIIIA domain is involved in adverse remodeling after tissue injury (Arslan et al., 2011; Hirshoren et al., 2013; Kohan et al., 2010; Muro et al., 2008). In MS lesions, Fn assembles into aggregates, which is likely mediated by inflammatory factors. Astrocytes are an important source of Fn aggregates, and these Fn aggregates contribute to remyelination failure (Stoffels et al., 2013a). Since our findings indicate that aFn is a nonessential element for remyelination, developing therapeutic approaches that remove aggregates of Fn from MS lesions represent a legitimate translational objective.

## References

[glia22748-bib-0001] Arnett HA , Fancy SP , Alberta JA , Zhao C , Plant SR , Kaing S , Raine CS , Rowitch DH , Franklin RJM , Stiles CD . 2004 bHLH transcription factor Olig1 is required to repair demyelinated lesions in the CNS. Science 306:2111–2115. 15604411 10.1126/science.1103709

[glia22748-bib-0002] Arslan F , Smeets MB , Riem Vis PW , Karper JC , Quax PH , Bongartz LG , Peters JH , Hoefer IE , Doevendans PA , Pasterkamp G , de Kleijn DP . 2011 Lack of fibronectin‐EDA promotes survival and prevents adverse remodeling and heart function deterioration after myocardial infarction. Circ Res 108:582–592. 21350212 10.1161/CIRCRESAHA.110.224428

[glia22748-bib-0003] Balza E , Sassi F , Ventura E , Parodi A , Fossati S , Blalock W , Carnemolla B , Castellani P , Zardi L , Borsi L . 2009 A novel human fibronectin cryptic sequence unmasked by the insertion of the angiogenesis‐associated extra type III domain B. Int J Cancer 125:751–758. 19479996 10.1002/ijc.24473

[glia22748-bib-0004] Baron W , Shattil SJ , ffrench‐Constant C . 2002 The oligodendrocyte precursor mitogen PDGF stimulates proliferation by activation of alpha(v)beta3 integrins. Embo J 21:1957–1966. 11953315 10.1093/emboj/21.8.1957 PMC125971

[glia22748-bib-0005] Bhat RV , Axt KJ , Fosnaugh JS , Smith KJ , Johnson KA , Hill DE , Kinzler KW , Baraban JM . 1996 Expression of the APC tumor suppressor protein in oligodendroglia. Glia 17:169–174. 8776583 10.1002/(SICI)1098-1136(199606)17:2<169::AID-GLIA8>3.0.CO;2-Y

[glia22748-bib-0006] Blakemore WF , Franklin RJM . 2008 Remyelination in experimental models of toxin‐induced demyelination. Curr Top Microbiol Immunol 318:193–212. 18219819 10.1007/978-3-540-73677-6_8

[glia22748-bib-0007] Blaschuk KL , Frost EE , ffrench‐Constant C . 2000 The regulation of proliferation and differentiation in oligodendrocyte progenitor cells by alphaV integrins. Development 127:1961–1969. 10751184 10.1242/dev.127.9.1961

[glia22748-bib-0008] Bsibsi M , Nomden A , van Noort JM , Baron W . 2012 Toll‐like receptors 2 and 3 agonists differentially affect oligodendrocyte survival, differentiation, and myelin membrane formation. J Neurosci Res 90:388–398. 21971760 10.1002/jnr.22767

[glia22748-bib-0009] Buttery PC , ffrench‐Constant C . 1999 Laminin‐2/integrin interactions enhance myelin membrane formation by oligodendrocytes. Mol Cell Neurosci 14:199–212. 10576890 10.1006/mcne.1999.0781

[glia22748-bib-0010] Cahoy JD , Emery B , Kaushal A , Foo LC , Zamanian JL , Christopherson KS , Xing Y , Lubischer JL , Krieg PA , Krupenko SA , Thompson WJ , Barres BA . 2008 A transcriptome database for astrocytes, neurons, and oligodendrocytes: A new resource for understanding brain development and function. J Neurosci 28:264–278. 18171944 10.1523/JNEUROSCI.4178-07.2008 PMC6671143

[glia22748-bib-0011] Chari DM , Zhao C , Kotter MR , Blakemore WF , Franklin RJM . 2006 Corticosteroids delay remyelination of experimental demyelination in the rodent central nervous system. J Neurosci Res 83:594–605. 16429447 10.1002/jnr.20763

[glia22748-bib-0012] Colognato H , Baron W , Avellana‐Adalid V , Relvas JB , Baron‐Van Evercooren A , Georges Labouesse E , ffrench‐Constant C . 2002 CNS integrins switch growth factor signalling to promote target‐dependent survival. Nat Cel Biol 11:833–841. 10.1038/ncb865 12379866

[glia22748-bib-0013] Duncan ID , Brower A , Kondo Y , Curlee JF Jr , Schultz RD . 2009 Extensive remyelination of the CNS leads to functional recovery. Proc Natl Acad Sci USA 106:6832–6836. 19342494 10.1073/pnas.0812500106 PMC2672502

[glia22748-bib-0014] Fancy SP , Baranzini SE , Zhao C , Yuk DI , Irvine KA , Kaing S , Sanai N , Franklin RJM , Rowitch DH . 2009 Dysregulation of the wnt pathway inhibits timely myelination and remyelination in the mammalian CNS. Genes Dev 23:1571–1585. 19515974 10.1101/gad.1806309 PMC2704469

[glia22748-bib-0015] Fancy SP , Zhao C , Franklin RJM . 2004 Increased expression of Nkx2.2 and Olig2 identifies reactive oligodendrocyte progenitor cells responding to demyelination in the adult CNS. Mol Cell Neurosci 27:247–254. 15519240 10.1016/j.mcn.2004.06.015

[glia22748-bib-0016] ffrench‐Constant C , Van de Water L , Dvorak HF , Hynes RO . 1989 Reappearance of an embryonic pattern of fibronectin splicing during wound healing in the adult rat. J Cell Biol 109:903–914. 2760116 10.1083/jcb.109.2.903 PMC2115730

[glia22748-bib-0017] Ford CC , Ceckler TL , Karp J , Herndon RM . 1990 Magnetic resonance imaging of experimental demyelinating lesions. Magn Reson Med 14:461–481. 2355829 10.1002/mrm.1910140305

[glia22748-bib-0018] Franklin RJM , ffrench‐Constant C . 2008 Remyelination in the CNS: From biology to therapy. Nat Rev Neurosci 9:839–855. 18931697 10.1038/nrn2480

[glia22748-bib-0019] Franklin RJM , ffrench‐Constant C , Edgar JM , Smith KJ . 2012 Neuroprotection and repair in multiple sclerosis. Nat Rev Neurol 8:624–634. 23026979 10.1038/nrneurol.2012.200

[glia22748-bib-0020] George EL , Georges‐Labouesse EN , Patel‐King RS , Rayburn H , Hynes RO . 1993 Defects in mesoderm, neural tube and vascular development in mouse embryos lacking fibronectin. Development 119:1079–1091. 8306876 10.1242/dev.119.4.1079

[glia22748-bib-0021] Gerdes J , Schwab U , Lemke H , Stein H . 1983 Production of a mouse monoclonal antibody reactive with a human nuclear antigen associated with cell proliferation. Int J Cancer 31:13–20. 6339421 10.1002/ijc.2910310104

[glia22748-bib-0022] Hibbits N , Yoshino J , Le TQ , Armstrong RC . 2012 Astrogliosis during acute and chronic cuprizone demyelination and implications for remyelination. ASN Neuro 30:393–408. 23025787 10.1042/AN20120062 PMC3483617

[glia22748-bib-0023] Hinks GL , Franklin RJM . 1999 Distinctive patterns of PDGF‐A, FGF‐2, IGF‐I, and TGF‐b1 gene expression during remyelination of experimentally‐induced spinal cord demyelination. Mol Cell Neurosci 14:153–168. 10532806 10.1006/mcne.1999.0771

[glia22748-bib-0024] Hirrlinger PG , Scheller A , Braun C , Hirrlinger J , Kirchhoff F . 2006 Temporal control of gene recombination in astrocytes by transgenic expression of the tamoxifen‐inducible DNA recombinase variant CreERT2. Glia 54:11–20. 16575885 10.1002/glia.20342

[glia22748-bib-0025] Hirshoren N , Kohan M , Assayag M , Neuman T , Vernea F , Muro A , Eliashar R , Berkman N . 2013 Extra domain‐A fibronectin is necessary for the development of nasal remodeling in chronic allergen‐induced rhinitis. Ann Allergy Asthma Immunol 110:322–327. 23622001 10.1016/j.anai.2013.03.002

[glia22748-bib-0026] Hu J , Deng L , Wang X , Xu XM . 2009 Effects of extracellular matrix molecules on the growth properties of oligodendrocyte progenitor cells in vitro. J Neurosci Res 87:2854–2862. 19472225 10.1002/jnr.22111

[glia22748-bib-0027] Ibanez C , Shields SA , El‐Etr M , Baulieu EE , Schumacher M , Franklin RJM . 2004 Systemic progesterone administration results in a partial reversal of the age‐associated decline in CNS remyelination following toxin‐induced demyelination in male rats. Neuropathol Appl Neurobiol 30:80–89. 14720179 10.1046/j.0305-1846.2003.00515.x

[glia22748-bib-0028] Imai Y , Ibata I , Ito D , Ohsawa K , Kohsaka S . 1996 A novel gene iba1 in the major histocompatibility complex class III region encoding an EF hand protein expressed in a monocytic lineage. Biochem Biophys Res Commun 224:855–862. 8713135 10.1006/bbrc.1996.1112

[glia22748-bib-0029] Jarnagin WR , Rockey DC , Koteliansky VE , Wang SS , Bissell DM . 1994 Expression of variant fibronectins in wound healing: Cellular source and biological activity of the EIIIA segment in rat hepatic fibrogenesis. J Cell Biol 127:2037–2048. 7806580 10.1083/jcb.127.6.2037 PMC2120289

[glia22748-bib-0030] Kohan M , Muro AF , White ES , Berkman N . 2010 EDA‐containing cellular fibronectin induces fibroblast differentiation through binding to alpha4beta7 integrin receptor and MAPK/Erk 1/2‐dependent signaling. FASEB J 24:4503–4512. 20643910 10.1096/fj.10-154435 PMC6188222

[glia22748-bib-0031] Kondo T , Raff M . 2004 Chromatin remodeling and histone modification in the conversion of oligodendrocyte precursors to neural stem cells. Genes Dev 18:2963–2972. 15574597 10.1101/gad.309404 PMC534656

[glia22748-bib-0032] Lau LW , Keough MB , Haylock‐Jacobs S , Cua R , Doring A , Sloka S , Stirling DP , Rivest S , Yong VW . 2012 Chondroitin sulfate proteoglycans in demyelinated lesions impair remyelination. Ann Neurol 72:419–432. 23034914 10.1002/ana.23599

[glia22748-bib-0033] Leone DP , Genoud S , Atanasoski S , Grausenburger R , Berger P , Metzger D , Macklin WB , Chambon P , Suter U . 2003 Tamoxifen‐inducible glia‐specific cre mice for somatic mutagenesis in oligodendrocytes and schwann cells. Mol Cell Neurosci 22:430–440. 12727441 10.1016/s1044-7431(03)00029-0

[glia22748-bib-0034] Liao YF , Gotwals PJ , Koteliansky VE , Sheppard D , Van De Water L . 2002 The EIIIA segment of fibronectin is a ligand for integrins alpha9beta1 and alpha4beta 1 providing a novel mechanism for regulating cell adhesion by alternative splicing. J Biol Chem 277:14467–14474. 11839764 10.1074/jbc.M201100200

[glia22748-bib-0035] Liao YF , Wieder KG , Classen JM , Van De Water L . 1999 Identification of two amino acids within the EIIIA (ED‐A) segment of fibronectin constituting the epitope for two function‐blocking monoclonal antibodies. J Biol Chem 274:17876–17884. 10364233 10.1074/jbc.274.25.17876

[glia22748-bib-0036] Liesi P , Dahl D , Vaheri A . 1983 Laminin is produced by early rat astrocytes in primary culture. J Cell Biol 96:920–924. 6339524 10.1083/jcb.96.3.920 PMC2112419

[glia22748-bib-0037] Liesi P , Kaakkola S , Dahl D , Vaheri A . 1984 Laminin is induced in astrocytes of adult brain by injury. Embo J 3:683–686. 6370690 10.1002/j.1460-2075.1984.tb01867.x PMC557407

[glia22748-bib-0038] Lovatt D , Sonnewald U , Waagepetersen HS , Schousboe A , He W , Lin JH , Han X , Takano T , Wang S , Sim FJ , Goldman SA , Nedergaard M . 2007 The transcriptome and metabolic gene signature of protoplasmic astrocytes in the adult murine cortex. J Neurosci 27:12255–12266. 17989291 10.1523/JNEUROSCI.3404-07.2007 PMC6673251

[glia22748-bib-0039] Livak KJ , Schmittgen TD . 2001 Analysis of relative gene expression data using real‐time quantitative PCR and the 2(‐Delta Delta C(T)) Method. Methods 25:402–408. 11846609 10.1006/meth.2001.1262

[glia22748-bib-0040] Maier O , van der Heide T , van Dam AM , Baron W , de Vries H , Hoekstra D . 2005 Alteration of the extracellular matrix interferes with raft association of neurofascin in oligodendrocytes. Potential significance for multiple sclerosis? Mol Cell Neurosci 28:390–401. 15691718 10.1016/j.mcn.2004.09.012

[glia22748-bib-0041] Manabe R , Ohe N , Maeda T , Fukuda T , Sekiguchi K . 1997 Modulation of cell‐adhesive activity of fibronectin by the alternatively spliced EDA segment. J Cell Biol 139:295–307. 9314547 10.1083/jcb.139.1.295 PMC2139828

[glia22748-bib-0042] Manabe R , Ohe N , Sekiguchi K . 1999 Alternatively spliced EDA segment regulates fibronectin‐dependent cell cycle progression and mitogenic signal transduction. J Biol Chem 274:5919–5924. 10026216 10.1074/jbc.274.9.5919

[glia22748-bib-0043] McCarthy KD , de Vellis J . 1980 Preparation of separate astroglial and oligodendroglial cell cultures from rat cerebral tissue. J Cell Biol 85:890–902. 6248568 10.1083/jcb.85.3.890 PMC2111442

[glia22748-bib-0044] Milner R , Edwards G , Streuli C , ffrench‐Constant C . 1996 A role in migration for the alpha V beta 1 integrin expressed on oligodendrocyte precursors. J Neurosci 16:7240–7252. 8929432 10.1523/JNEUROSCI.16-22-07240.1996 PMC6578950

[glia22748-bib-0045] Milner R , ffrench‐Constant C . 1994 A developmental analysis of oligodendroglial integrins in primary cells: Changes in alpha v‐associated beta subunits during differentiation. Development 120:3497–3506. 7821217 10.1242/dev.120.12.3497

[glia22748-bib-0046] Moretti FA , Chauhan AK , Iaconcig A , Porro F , Baralle FE , Muro AF . 2007 A major fraction of fibronectin present in the extracellular matrix of tissues is plasma‐derived. J Biol Chem 282:28057–28062. 17644525 10.1074/jbc.M611315200

[glia22748-bib-0047] Muro AF , Moretti FA , Moore BB , Yan M , Atrasz RG , Wilke CA , Flaherty KR , Martinez FJ , Tsui JL , Sheppard D , Baralle FE , Toews GB , White ES . 2008 An essential role for fibronectin extra type III domain A in pulmonary fibrosis. Am J Respir Crit Care Med 177:638–645. 18096707 10.1164/rccm.200708-1291OC PMC2267338

[glia22748-bib-0048] Nickeleit V , Kaufman AH , Zagachin L , Dutt JE , Foster CS , Colvin RB . 1996 Healing corneas express embryonic fibronectin isoforms in the epithelium, subepithelial stroma, and endothelium. Am J Pathol 149:549–558. 8701994 PMC1865294

[glia22748-bib-0049] Olsen AL , Sackey BK , Marcinkiewicz C , Boettiger D , Wells RG . 2012 Fibronectin extra domain‐A promotes hepatic stellate cell motility but not differentiation into myofibroblasts. Gastroenterology 142:928–937. 22202457 10.1053/j.gastro.2011.12.038 PMC3321084

[glia22748-bib-0050] Ou J , Deng J , Wei X , Xie G , Zhou R , Yu L , Liang H . 2013 Fibronectin extra domain A (EDA) sustains CD133(+)/CD44(+) subpopulation of colorectal cancer cells. Stem Cell Res 11:820–833. 23811539 10.1016/j.scr.2013.05.009 PMC3917514

[glia22748-bib-0051] Patrikios P , Stadelmann C , Kutzelnigg A , Rauschka H , Schmidbauer M , Laursen H , Sorensen PS , Bruck W , Lucchinetti C , Lassmann H . 2006 Remyelination is extensive in a subset of multiple sclerosis patients. Brain 129:3165–3172. 16921173 10.1093/brain/awl217

[glia22748-bib-0052] Paul JI , Schwarzbauer JE , Tamkun JW , Hynes RO . 1986 Cell‐type‐specific fibronectin subunits generated by alternative splicing. J Biol Chem 261:12258–12265. 3528152

[glia22748-bib-0053] Pickford AR , Campbell ID . 2004 NMR studies of modular protein structures and their interactions. Chem Rev 104:3557–3566. 15303827 10.1021/cr0304018

[glia22748-bib-0054] Sakai T , Johnson KJ , Murozono M , Sakai K , Magnuson MA , Wieloch T , Cronberg T , Isshiki A , Erickson HP , Fassler R . 2001 Plasma fibronectin supports neuronal survival and reduces brain injury following transient focal cerebral ischemia but is not essential for skin‐wound healing and hemostasis. Nat Med 7:324–330. 11231631 10.1038/85471

[glia22748-bib-0055] Satoh JI , Tabunoki H , Yamamura T . 2009 Molecular network of the comprehensive multiple sclerosis brain‐lesion proteome. Mult Scler 15:531–541. 19389748 10.1177/1352458508101943

[glia22748-bib-0056] Schwarzbauer JE , Patel RS , Fonda D , Hynes RO . 1987 Multiple sites of alternative splicing of the rat fibronectin gene transcript. EMBO J 6:2573–2580. 2445560 10.1002/j.1460-2075.1987.tb02547.x PMC553677

[glia22748-bib-0057] Shen S , Sandoval J , Swiss VA , Li J , Dupree J , Franklin RJM , Casaccia‐Bonnefil P . 2008 Age‐dependent epigenetic control of differentiation inhibitors is critical for remyelination efficiency. Nat Neurosci 11:1024–1034. 19160500 10.1038/nn.2172 PMC2656679

[glia22748-bib-0059] Siskova Z , Baron W , de Vries H , Hoekstra D . 2006 Fibronectin impedes “myelin” sheet‐directed flow in oligodendrocytes: A role for a beta1 integrin‐mediated PKC signaling pathway in vesicular trafficking. Mol Cell Neurosci 33:150–159. 16935002 10.1016/j.mcn.2006.07.001

[glia22748-bib-0060] Siskova Z , Yong VW , Nomden A , van Strien M , Hoekstra D , Baron W . 2009 Fibronectin attenuates process outgrowth in oligodendrocytes by mislocalizing MMP‐9 activity. Mol Cell Neurosci 42:234–242. 19607919 10.1016/j.mcn.2009.07.005

[glia22748-bib-0061] Sobel RA , Mitchell ME . 1989 Fibronectin in multiple sclerosis lesions. Am J Pathol 135:161–168. 2528301 PMC1880224

[glia22748-bib-0062] Stenzel D , Lundkvist A , Sauvaget D , Busse M , Graupera M , van der Flier A , Wijelath ES , Murray J , Sobel M , Costell M , Takahashi S , Fassler R , Yamaguchi Y , Gutmann DH , Hynes RO , Gerhardt H . 2011 Integrin‐dependent and ‐independent functions of astrocytic fibronectin in retinal angiogenesis. Development 138:4451–4463. 21880786 10.1242/dev.071381 PMC3177315

[glia22748-bib-0063] Stidworthy MF , Genoud S , Li WW , Leone DP , Mantei N , Suter U , Franklin RJM . 2004 Notch1 and Jagged1 are expressed after CNS demyelination, but are not a major rate‐determining factor during remyelination. Brain 127:1928–1941. 15289265 10.1093/brain/awh217

[glia22748-bib-0064] Stoffels JMJ , de Jonge JC , Stancic M , Nomden A , van Strien ME , Ma D , Siskova Z , Maier O , ffrench‐Constant C , Franklin RJM , Hoekstra D , Zhao C , Baron W . 2013a Fibronectin aggregation in multiple sclerosis lesions impairs remyelination. Brain 136:116–131. 23365094 10.1093/brain/aws313

[glia22748-bib-0065] Stoffels JMJ , Zhao C , Baron W . 2013b Fibronectin in tissue regeneration: Timely disassembly of the scaffold is necessary to complete the build. Cell Mol Life Sci 70:4243–4253. 23756580 10.1007/s00018-013-1350-0 PMC11113129

[glia22748-bib-0066] Sun XJ , Fa PP , Cui ZW , Xia Y , Sun L , Li ZS , Tang AF , Gui YT , Cai ZM . 2014 The EDA‐containing cellular fibronectin induces epithelial mesenchymal transition in lung cancer cells through integrin alpha9beta1‐mediated activation of PI3‐K /Akt and Erk1/2. Carcinogenesis 35:184–191. 10.1093/carcin/bgt276 23929437

[glia22748-bib-0067] To WS , Midwood KS . 2011 Plasma and cellular fibronectin: Distinct and independent functions during tissue repair. Fibrogenesis Tissue Repair 4:21. 21923916 10.1186/1755-1536-4-21 PMC3182887

[glia22748-bib-0068] Ulrich MM , Janssen AM , Daemen MJ , Rappaport L , Samuel JL , Contard F , Smits JF , Cleutjens JP . 1997 Increased expression of fibronectin isoforms after myocardial infarction in rats. J Mol Cell Cardiol 29:2533–2543. 9299376 10.1006/jmcc.1997.0486

[glia22748-bib-0069] van Horssen J , Bo L , Vos CM , Virtanen I , de Vries HE . 2005 Basement membrane proteins in multiple sclerosis‐associated inflammatory cuffs: Potential role in influx and transport of leukocytes. J Neuropathol Exp Neurol 64:722–729. 16106221 10.1097/01.jnen.0000173894.09553.13

[glia22748-bib-0070] Ventura E , Sassi F , Parodi A , Balza E , Borsi L , Castellani P , Carnemolla B , Zardi L . 2010 Alternative splicing of the angiogenesis associated extra‐domain B of fibronectin regulates the accessibility of the B‐C loop of the type III repeat 8. PLoS One 5:e9145. 20161770 10.1371/journal.pone.0009145 PMC2818841

[glia22748-bib-0071] von Au A , Vasel M , Kraft S , Sens C , Hackl N , Marx A , Stroebel P , Hennenlotter J , Todenhofer T , Stenzl A , Schott S , Sinn HP , Wetterwald A , Bermejo JL , Cecchini MG , Nakchbandi IA . 2013 Circulating fibronectin controls tumor growth. Neoplasia 15:925–938. 23908593 10.1593/neo.13762 PMC3730044

[glia22748-bib-0072] Watanabe M , Hadzic T , Nishiyama A . 2004 Transient upregulation of Nkx2.2 expression in oligodendrocyte lineage cells during remyelination. Glia 46:311–322. 15048854 10.1002/glia.20006

[glia22748-bib-0073] Xia P , Culp LA . 1995 Adhesion activity in fibronectin's alternatively spliced domain EDa (EIIIA): Complementarity to plasma fibronectin functions. Exp Cell Res 217:517–527. 7698252 10.1006/excr.1995.1117

[glia22748-bib-0074] Zawadzka M , Rivers LE , Fancy SP , Zhao C , Tripathi R , Jamen F , Young K , Goncharevich A , Pohl H , Rizzi M , Rowitch DH , Kessaris N , Suter U , Richardson WD , Franklin RJM . 2010 CNS‐resident glial progenitor/stem cells produce schwann cells as well as oligodendrocytes during repair of CNS demyelination. Cell Stem Cell 6:578–590. 20569695 10.1016/j.stem.2010.04.002 PMC3856868

[glia22748-bib-0075] Zhao C , Fancy SP , ffrench‐Constant C , Franklin RJM . 2008 Osteopontin is extensively expressed by macrophages following CNS demyelination but has a redundant role in remyelination. Neurobiol Dis 31:209–217. 18539470 10.1016/j.nbd.2008.04.007

[glia22748-bib-0076] Zhao C , Fancy SP , Franklin RJM , ffrench‐Constant C . 2009 Up‐regulation of oligodendrocyte precursor cell alphaV integrin and its extracellular ligands during central nervous system remyelination. J Neurosci Res 87:3447–3455. 19739252 10.1002/jnr.22231

[glia22748-bib-0077] Zhao C , Li WW , Franklin RJM . 2006 Differences in the early inflammatory responses to toxin‐induced demyelination are associated with the age‐related decline in CNS remyelination. Neurobiol Aging 27:1298–1307. 16051398 10.1016/j.neurobiolaging.2005.06.008

